# Advancing Comprehensive Stroke Care—From Acute Recovery to Long-Term Wellbeing

**DOI:** 10.3390/healthcare13101193

**Published:** 2025-05-20

**Authors:** Tharshanah Thayabaranathan, Dominique A. Cadilhac

**Affiliations:** 1 Stroke and Ageing Research Group, Department of Medicine, School of Clinical Sciences at Monash Health, Monash University, Clayton, VIC 3168, Australia; dominique.cadilhac@monash.edu; 2 Stroke Group, Florey Institute of Neuroscience and Mental Health, University of Melbourne, Heidelberg, VIC 3084, Australia

## Introduction

Stroke is one of the most complex diseases of our time; it impacts individuals across many facets of functioning, depending on the areas of the brain that have been damaged. Despite substantial advances in acute management, the global burden of stroke is increasing, especially in low- and middle-income countries (LMICs), where stroke occurs at younger ages and leads to more years lived with disability [1,2]. It has been estimated that nearly 89% of stroke-related disability-adjusted life years (DALYs) now occur in LMICs [1,2]. This disproportionate burden in LMICs contrasts with high-income countries, which increasingly face challenges related to ageing populations [3]. Consequently, there is an urgent need to prevent stroke, and to also embrace the full spectrum of treatment and recovery care including rehabilitation, avoiding complications, and providing support for mental health. Since stroke can often be fatal or increase the risk of death, appropriate palliative care support is also a priority.

The Special Issue “Stroke and Ageing” brings together ten articles from different regions that cover the spectrum of advancing practice to meet healthcare needs after stroke. Each article contributes to the growing body of evidence supporting an integrated, person-centred, and lifespan-oriented approach to stroke care. Drawing on insights from Australia, China, South Korea, and the United Kingdom, the articles reflect diverse healthcare contexts and patient populations and are listed below:

1. Lee, J.H. Analysis of Grip Strength Thresholds for Stroke Management and Prevention in South Korean Older Adults. *Healthcare* **2025**, *13*, 781.
2. Carey, L.M.; Cahill, L.S.; Blennerhassett, J.M.; Nilsson, M.; Lannin, N.A.; Thijs, V.; Hillier, S.; Cadilhac, D.A.; Donnan, G.A.; Morris, M.E.; et al. A Network of Sites and Upskilled Therapists to Deliver Best-Practice Stroke Rehabilitation of the Arm: Protocol for a Knowledge Translation Study. *Healthcare* **2023**, *11*, 3080.
3. Marsden, D.L.; Boyle, K.; Birnie, J.; Buzio, A.; Dizon, J.; Dunne, J.; Greensill, S.; Hill, K.; Lever, S.; Minett, F.; et al. Improving Practice for Urinary Continence Care on Adult Acute Medical and Rehabilitation Wards: A Multi-Site, Co-Created Implementation Study. *Healthcare* **2023**, *11*, 1241.
4. Lightbody, C.E.; Patel, K.; Holland, E.-J.; Sutton, C.J.; Brown, C.; Tishkovskaya, S.V.; Bowen, A.; Read, J.; Thomas, S.; Roberts, T.; et al. Accelerating the Delivery of Psychological Therapies After Stroke: A Feasibility Stepped-Wedge Cluster Randomised Controlled Trial. *Healthcare* **2025**, *13*, 824824.
5. Baker, C.; Thomas, S.; Tjokrowijoto, P.; Ryan, B.; Kneebone, I.; Stolwyk, R. Aphasia Depression and Psychological Therapy (ADaPT): Perspectives of People with Post-Stroke Aphasia on Participating in a Modified Cognitive Behavioral Therapy. *Healthcare* **2024**, *12*, 771.
6. Hunter, S.; Vogel, K.; O’Leary, S.; Blennerhassett, J.M. Evaluating Feasibility of a Secondary Stroke Prevention Program. *Healthcare* **2023**, *11*, 2673.
7. Rehman, S.; Barker, S.; Jose, K.; Callisaya, M.; Castley, H.; Schultz, M.G.; Moore, M.N.; Simpson, D.B.; Peterson, G.M.; Gall, S. Co-Designed Cardiac Rehabilitation for the Secondary Prevention of Stroke (CARESS): A Pilot Program Evaluation. *Healthcare* **2024**, *12*, 776.
8. Wang, X.; Jiang, H.; Zhao, Z.; Kevine, N.T.; An, B.; Ping, Z.; Lin, B.; Zhang, Z. Mediation Role of Behavioral Decision-Making Between Self-Efficacy and Self-Management Among Elderly Stroke Survivors in China: Cross-Sectional Study. *Healthcare* **2025**, *13*, 704.
9. Wong, D.; Sanders, L.M.; Beauchamp, A.; Formby, C.; Smith, E.E.; Hansen, C.; McKinley, K.; De Jongh, K.; Borschmann, K. “When the Word Is too Big, It’s Just too Hard”: Stroke Survivors’ Perspectives About Health Literacy and Delivery of Health Information. *Healthcare* **2025**, *13*, 541.
10. Lightbody, C.E.; Gordon, C.; Burton, C.; Davidson, C.; Jenkinson, D.; Patel, A.S.; Petrie, F.J.; Rouncefield-Swales, A.; Sprigg, N.; Stewart, K.; et al. Prepare: Improving End-of-Life Care Practice in Stroke Care: Insights from a National Survey and Semi-Structured Interviews. *Healthcare* **2025**, *13*, 848.

The article by Lee illustrates the use of cross-sectional, national survey data to identify sex-specific thresholds for absolute and relative grip strength as predictors of stroke risk in older Korean adults (article 1). The findings from the study may support the potential use of grip strength as a simple, scalable tool for stroke risk stratification. However, validation in longitudinal cohort studies is needed. The other nine articles cover four main themes: *physical rehabilitation, psychological health, secondary prevention,* and *end-of-life care* for people with stroke. Collectively, these articles underscore a common conclusion that stroke care must encompass long-term functional recovery, quality of life, and overall wellbeing across the remaining lifespan. A summary of the four main themes covered in this Special Issue is provided in the next sections.

## 1. Physical Rehabilitation

Rehabilitation is the single most effective intervention for improving quality of life after stroke. However, it is estimated that only 30–50% of patients receive guideline-recommended physical rehabilitation, often due to geographic, financial, or staffing constraints [4,5].

The authors of two implementation studies included in this issue provide examples of approaches to improve access to evidence-based rehabilitation after stroke. Carey et al. present their protocol for an innovative, multicentre knowledge translation project to increase access to upper limb rehabilitation by upskilling and credentialing occupational therapists or physiotherapists and using a network model to increase reach across Australia (article 2). Grounded in implementation science theory, their project is designed to ensure therapists know what should be delivered while establishing a model of care to promote consistent adoption in real-world settings. The target population are those with loss of body sensation, which can affect one in two people after stroke [6]. This example illustrates to readers how to design studies to increase the adoption of new, effective models of care and the importance of establishing partnerships with consumers, policy-makers, clinicians, healthcare organisations, and researchers.

In the article by Marsden et al., the focus is on improving the detection and management of urinary incontinence in acute hospitals and in-patient rehabilitation settings (article 3). Urinary incontinence is a common complication after stroke, affecting more than half of stroke patients within the first month, 38% at one year, and 17% in the long term [7]. The authors of this multi-site, pre–post intervention study aimed to increase the adoption of a co-designed urinary continence care intervention known as SCAMP. Conducted in 15 wards among 12 Australian hospitals, the knowledge translation intervention significantly improved assessment and care planning for patients with incontinence; overall, the odds of receiving assessments and management plans for urinary incontinence increased 4-fold. Given the psychological, physical, and social distress associated with incontinence [7], the study contributes meaningfully to supporting patient dignity and autonomy. The research provides another example of the importance of co-design, whereby project leads and implementation champions (mainly nurses), clinical experts in continence care, and researchers ensured clinical relevance to the settings for adoption. The use of implementation science theory is also exemplified for readers.

Globally, as noted by Feigin et al. and the World Health Organization, rehabilitation must become a ‘universal health service’ accessible to all, not just those with private insurance or urban access [8,9]. The articles by Carey et al. and Marsden et al. provide foundational models and adaptable implementation frameworks designed for scalability across diverse healthcare systems.

## 2. Psychological Health

Psychological distress, including depression, anxiety, and emotional dysregulation, affects up to 60% of survivors of stroke, yet it is one of the most under-addressed aspects of post-stroke care [10,11]. In this Special Issue, there are two complementary studies providing insights on novel approaches to addressing psychological problems after stroke.

Lightbody et al. (the ADOPTS trial) conducted a feasibility stepped-wedge randomised controlled trial co-designed to embed psychological support pathways within four English National Health Service (NHS) stroke units (article 4). Their approach included mood screening algorithms, staff training, supervision structures, and integration with an existing programme, NHS Talking Therapies. The intervention was found to improve staff confidence, and the authors were also able to illustrate how psychological care can be systematised and scaled. An important lesson was to allow sufficient time for staff training, since time to release clinical staff for training can be challenging.

Baker et al. conducted qualitative interviews with survivors of stroke who also had aphasia, a communication disorder that can result in being excluded from mental health services (article 5). Their evaluation of ADaPT, a modified Cognitive–Behavioural Therapy programme tailored through visual supports and simplified language, revealed benefits to participants in mood regulation, communication, and self-acceptance. The programme was delivered through both telehealth and in-person modes, allowing for flexibility in delivery and accessibility.

The World Stroke Organization (WSO) and authors such as Ignacio et al. have emphasised that post-stroke depression remains a leading determinant of poor recovery, reintegration, and survival [2,11]. To address this, psychological interventions must be inclusive, proactive, and embedded within early discharge and long-term follow-up care.

## 3. Secondary Prevention

Globally, over 25% of strokes are recurrent, and nearly 80% of these could be prevented through risk factor modification [1,12]. Recurrent strokes are associated with greater severity and higher mortality rates compared to first-ever stroke events [13]. There is an urgent need to provide support for people after stroke to avoid another event through the use of medications, e.g., to lower blood pressure, and lifestyle behaviour change, such as improving diet and increasing exercise. Patient adherence to lifelong prevention strategies remains a major challenge. For example, 21% of survivors of stroke discontinue their blood pressure-lowering medications within the first year [14]. In this Special Issue, we include two articles with a focus on new programmes to increase support for secondary stroke prevention.

Hunter et al. piloted a 12-week secondary prevention programme combining supervised exercise, education, and telehealth coaching (article 6). Their results, obtained from 37 participants as part of a non-randomised feasibility study, provided evidence of improvements in modifiable risk factors and physical fitness. The intervention was highly acceptable; almost all ‘felt safe to exercise’ and ‘would recommend the programme to others’.

In the second non-randomised feasibility (pilot) trial by Rehman et al., the intervention was based on adapting a cardiac rehabilitation programme available in Australia. The programme was co-designed for survivors of stroke as a community-based model (article 7). The 10 participants improved their functional capacity and reported less fatigue, with strong indications of behavioural engagement. The authors noted various implementation challenges, including the division of care between state and federally funded programmes and services within the Australian context.

Overall, these studies align with the growing global movement towards providing comprehensive, community-delivered secondary prevention. The WSO and the World Heart Federation have each called for an integration of stroke and cardiac prevention models and for using shared infrastructure and health coaches, especially in rural and resource-constrained settings [15,16]. Another essential feature is the need to tailor and individualise programmes to ensure greater success.

An important aspect for adopting and changing behaviour includes a person’s self-efficacy and ability to self-manage their condition [17]. In the article by Wang et al., behavioural decision-making was found to mediate the relationship between self-efficacy (the ability to organise and execute action processes to achieve behavioural goals) and self-management (article 8). This research was undertaken with 291 elderly survivors of stroke from Henan Province, China. This suggests that simply providing education to patients after a stroke is not enough. Health professionals must also help patients develop the cognitive and behavioural skills needed to apply knowledge, assess risks, and sustain healthy behaviours that support recovery.

An essential component to support self-efficacy and self-management is understanding levels of health literacy as part of providing health education. In this Special Issue, Wong et al. discuss health literacy among people with stroke to illustrate the need for clinicians to tailor information and not make assumptions about patients’ prior knowledge (article 9). Using the Ophelia framework [18], they demonstrated that survivors with low health literacy would be less likely to understand their stroke, follow prevention advice, or feel confident in their care. The authors call for universal precautions in stroke communication and emphasise that every patient needs information tailored to their literacy and cognitive level, which is especially pertinent in multicultural and ageing societies.

## 4. End-of-Life Care

In many health systems, end-of-life care for stroke is under-resourced, under-researched, and inconsistently implemented. The study by Lightbody et al. offers a rare window into this domain through a UK-wide survey and interviews with multidisciplinary staff (article 10). Their findings are sobering: despite high stroke-related mortality and known prognostic uncertainty, only a minority of stroke units use stroke-specific tools to guide end-of-life decisions. The variability in practice was exacerbated by a lack of training and integration with palliative care services. These challenges reflect global trends. In LMICs, limited access to specialist care and cultural taboos around death further compound the problem. The solution, as Lightbody and colleagues argue, lies in pragmatic changes: stroke-adapted guidance, shared decision-making tools, training for all staff, and stronger integration with supportive care pathways.

Across the ten studies included in this Special Issue, four key principles emerge. First, a lifespan approach recognises stroke as a chronic condition requiring continuous support across physical, communication, sensory, incontinence, and psychological domains. Second, there is a strong focus on real-world implementation, with pragmatic designs such as stepped-wedge trials and co-design methods emphasising feasibility and sustainability. Third, a commitment to equity and inclusion is evident, with tailored interventions for people with aphasia, culturally diverse groups, and rural populations. Finally, the studies highlight the value of interdisciplinary collaboration, with examples such as integrated psychological support, multidisciplinary continence care, and telehealth coaching. Together, these principles underscore a shift toward holistic, patient-centred stroke care.

## Summary

Stroke is not solely a medical condition—it is a complex societal challenge. Its impact extends beyond mortality, encompassing lost productivity, increased care needs, and a long-term dependence on health and social systems. The ten studies presented in this Special Issue make it clear that the solutions may be within reach. We now possess the tools, knowledge, and evidence base required to transform stroke care. What remains is a collective will to act—to implement these strategies at scale, to embed them into systems, and to sustain them over time.

As editors of this Special Issue, we commend the authors for their scholarly rigour and for providing research that has clinical relevance and will lead to greater inclusion and accessibility to evidence-based programmes that support long-term wellbeing. The articles in this Special Issue represent a range of research methods, including co-design, use of implementation science theory, and feasibility trials, contributing to the growing need to find solutions that require complex system thinking and evaluation.
